# Analysis of Work Related Factors, Behavior, Well-Being Outcome, and Job Satisfaction of Workers of Emergency Medical Service: A Systematic Review

**DOI:** 10.3390/ijerph19116660

**Published:** 2022-05-30

**Authors:** Beatrice Thielmann, Julia Schnell, Irina Böckelmann, Heiko Schumann

**Affiliations:** Institute of Occupational Medicine, Medical Faculty, Otto von Guericke University, Leipziger Straße 44, 39120 Magdeburg, Germany; julia.schnell@st.ovgu.de (J.S.); irina.boeckelmann@med.ovgu.de (I.B.); heiko.schumann@med.ovgu.de (H.S.)

**Keywords:** workloads, rescue workers, emergency physicians, organization, health, stress

## Abstract

Background: The workloads of emergency medical service personnel (EMS) are varied. In the absence of recovery, health consequences can result. The aim of this review was to analyze the literature on the associations between psychosocial or physical work factors on one hand and the well-being outcomes and job satisfaction on the other hand. Methods: A systematic literature review examining the workloads, behavior, and well-being of EMS including emergency physicians, in accordance with the Preferred Reporting Items for Systematic Reviews and Meta-Analysis (PRISMA) statement for the reporting systematic reviews, was performed. The PubMed, Ovid Medline, Cochrane Library, Scopus, Web of Science, PsycINFO, Psyndex, and Embase electronic databases were used. Results: Thirty-three studies were included. These were divided into studies that predominantly focused on the behavior (6), stress and strain (22), and well-being (5) of EMS. Only four studies also examined emergency physicians. The studies indicated a high prevalence of psychological and physical stress factors. Burnout and posttraumatic stress disorders have been the most studied consequences of mismatched stress. The health status variable performs better in conjunction with higher qualifications. Age is not a protective variable in some studies. Conclusions: EMS workloads are varied and must be assessed on an individual basis. Studies on emergency physicians are needed. Organizational and personal measures must become the focus of health promotion and prevention in the workplace.

## 1. Introduction

The workloads of emergency service personnel (EMS) vary and include physical and mental strain [1,2]. It is necessary to separate operational stress such as daily stress due to the frequency of alerts about trivial emergencies, from stress due to life-saving operations or rare, extreme situations, and the general conditions related to emergency medical services and organizations [3].

The identification of workloads, the increased importance of psychological factors, and the resulting health consequences of occupational groups have reached levels of increased importance in research. Major stress factors at work include feelings of unfairness in the job, performance pressure, shift work, loss of control, low social support at work, and overtime work [4]. Emergency physicians demonstrate high levels of subjective and objective stress during alarm operations [5]. In addition, relationships between job satisfaction, life quality, and physical and mental health are well-known [6,7]. 

Workload can be defined as the balance between work-related stress and an individual’s response to that demand [8]. Work-related stress can be positive or negative as a short-term consequence. In the long-term, and not counteracting negative stresses, work-related stressors also affect one’s emotional state and are associated with depression, anxiety disorders, and cardiovascular diseases [9,10]. There are many stress models, although the recovery–stress concept according to Rohmert and Rutenfranz is the main one here, which describes the relationships between the work situation and the effect on the working person. The stress would be seen as quite value-neutral, but the stress is individually different [11,12]. This also means, for example, that social/family background and personality traits have an influence on stress [13].

The importance of occupational health in the emergency services is also becoming increasingly important in research. Therefore, surveys of the workloads and resources are very important for making comparisons with other studies from other countries, for learning from these studies, and for identifying the health promotion measures. The aim of this review was to systematically evaluate the literature on the associations between the measurements of the psychosocial or physical work factors on one hand, and the well-being outcomes and job satisfaction on the other, under the consideration of the work-related behavior of emergency ambulance personnel including emergency physicians. The first step was to look at the status quo regarding the above-mentioned topics for the emergency service personnel and emergency physicians (EP). Where possible, the occupational group differences were identified (comparison between EMS, EP). Hypothetically, we assumed that there were differences in the statements of the stresses and strains between the occupational groups. In addition, we suspected an impaired life satisfaction in stressed personnel and that behaviors influenced the outcomes.

## 2. Materials and Methods

We performed a systematic literature review that examined the strain, stress, behavior, and well-being among the emergency service personnel and emergency physicians in accordance with the Preferred Reporting Items for Systematic Reviews and Meta-Analysis (PRISMA) statement for the reporting of systematic reviews [14]. The following electronic databases were used: PubMed, Ovid Medline, Cochrane Library, Scopus, Web of Science, PsycINFO, Psyndex, and Embase. The search terms were defined as follows: (“ambulance service” OR “emergency medical service” OR “emergency services” OR “ambulance crew” OR “ambulance men” OR “ambulance driver” OR “emergency workers” OR “paramedic” OR “emergency medical technician” OR “paramedic service” OR “rescue service” OR “frontline workers” OR “emergency paramedic” OR “rescue workers” OR “emergency physician” OR “emergency doctor” OR “doctor on call” OR “helicopter doctor” OR “helicopter physician”) AND (“job satisfaction” AND (“wellbeing” OR “wellbeing” OR “wellbeing” OR “welfare” OR “physical comfort”) AND “strain” OR “stress” OR “working conditions” OR “psychological strain” OR “occupational stress”) AND (“work related behavior” OR “work related behavior” OR “work engagement” OR “subjective importance of work” OR “work-related ambition” OR “willingness to work until exhausted”) NOT “nurses”. A check of the reference lists of the included studies was also performed. We focused on the professional group of emergency service personnel with training as a paramedic or similar. These individuals work exclusively in the ambulance service, and not in the hospital emergency department. Therefore, “nurses” were excluded.

The inclusion criteria were having a full text in the English or German language; being published between 2000 and the deadline of 21 September 2021; using EMS employees with completed medical training; including more than 20 participants; having all psychosocial work factors and job characteristics derived from EMS self-reports or expert observations; having all well-being outcomes derived from individual EMS self-reports or expert evaluations; having all work-related behavior factors derived from EMS self-reports or expert observations, if applicable; using classification into different psychological groups; and using only humans as subjects. The exclusion criteria were using personnel working in hospital settings such as emergency departments; using first responders without completed medical training; having a number of subjects in each group <20; and other study types such as single-case studies, review articles, short communications, letters with insufficient information to analyze the results, guidelines, theses, dissertations, qualitative studies, scientific conference abstracts, studies on animals, and experimental studies. Studies that were not open access and could not be obtained through interlibrary loans or authors who did not respond to requests were also excluded.

The complete study protocol is available at Prospero at the following link: https://www.crd.york.ac.uk/PROSPERO/display_record.php?RecordID=279650 (accessed on 19 September 2021). 

The Citavi 6 reference manager (Swiss Academic Software, Wädenswil, Switzerland) was used. The articles found were transferred to Citavi and the duplicates were removed. The authors B.T. and J.S. independently reviewed the titles and abstracts according to the inclusion and exclusion criteria. The full text of each relevant article was then obtained. B.T. and J.S. again independently reviewed the full texts of these articles. Any discrepancies were clarified by discussion with the third and fourth reviewers (I.B. and H.S.).

The methodological quality of the included studies was evaluated using the Quality Assessment Tool for Quantitative Studies Dictionary [15]. This tool can also be used for one-time surveys or interviews. The EPHPP tool is based on the categories of selection bias, study design, confounders, blinding, data collection, withdrawals, and dropouts. Each of these elements was rated as strong, moderate, or weak for each article. Studies with two or more weak categories were rated as weak overall.

The questionnaire variables were collected from the studies. The following significances were used in the text to interpret the significance of the studies: * *p* < 0.05, ** *p* < 0.01, *** *p* < 0.001. The correlations were rated as follows: >0.3 moderate correlation and >0.5 strong correlation.

## 3. Results

### 3.1. General Results

**Figure 1:**
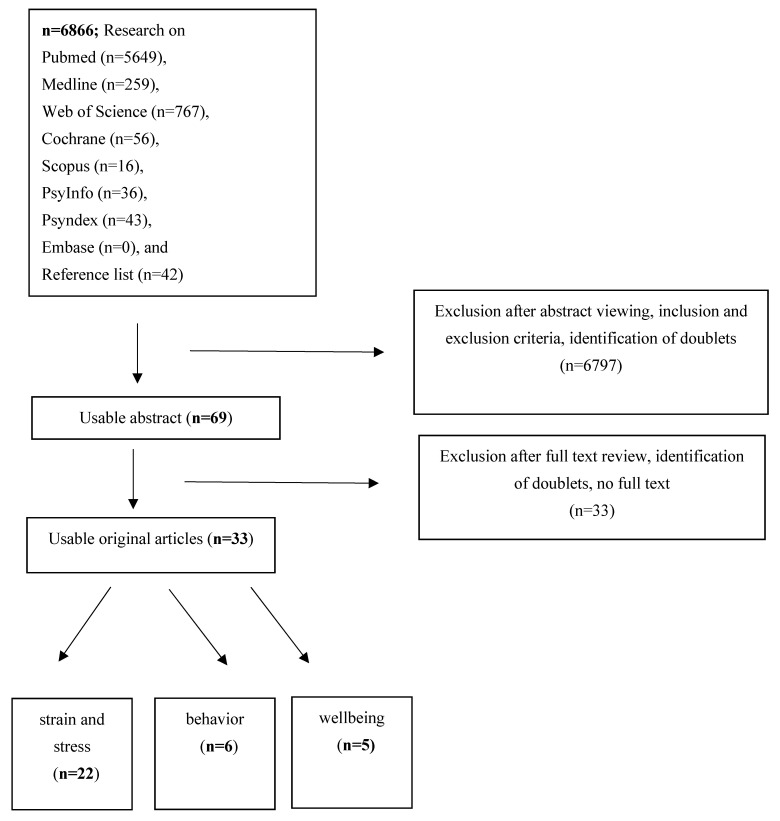
Flow chart of the review. *Note.* Some studies were assigned to two categories.

A total of 6866 citations were assessed. Duplicates were recognized by Citavi and not included again. After screening the titles and abstracts for the inclusion and exclusion criteria and adding from the reference lists, 69 full texts were screened for further eligibility. Of these, 26 texts did not fulfill the inclusion criteria, and no full text was available from an additional seven citations. Thus, 33 studies were included in the review. As expected, more studies (*n* = 22) were found on stress and strain [16,17,18,19,20,21,22,23,24,25,26,27,28,29,30,31,32,33,34,35,36,37,38] than on behavior (*n* = 6) [38,39,40,41,42,43] or well-being (*n* = 5) [41,44,45,46,47]. Some studies were assigned to two categories: behavior/stress and strain [38], well-being/stress and strain [28,35,46], or behavior/well-being [41]. The flowchart of the data analysis is shown in Figure 1.

In all of the studies, a total of 31,668 subjects were examined, ranging from 40 [32] to 17,522 subjects per study [31]. Three studies examined only men [24,36,38], two had more women than men [27,43], and one study did not report on gender [19]. There was no standardized professional classification. Included in the studies were paramedics (PM), emergency medical services professionals (EMS), emergency medical technicians (EMT), or drivers who worked in prehospital emergency services rather than hospital emergency departments. It remains unclear whether all job titles are the same in all countries. Only four studies considered emergency physicians [19,27,32,34]. The mean age of the subjects in the studies was between 30 and 40 years old. The studies were conducted worldwide. Most of the studies were performed in Europe [17,18,19,25,27,29,32,33,35,39,40,44,45,46,47]. Further studies came from Asia [20,23,26,36,38,41], Oceania [42], North America [21,22,28,30,31,43], South America [34], and Africa [24]. One study was international [22]. The meanings of each questionnaire are given in Supplementary File S1.

Various questionnaires were used. The Maslach Burnout Inventory (MBI) in different versions was used the most [16,19,24,27,29,36,37,38,40]. The focus on posttraumatic stress disorder (PTSD) was also examined in seven studies [17,18,21,23,25,26,28].

### 3.2. Interpretations of the Studies on Behavior

Six studies examined the behavior and coping of paramedics (Table 1) [38,39,40,41,42,43]. The results are shown in Supplementary File S2. For this topic, 1839 subjects were studied. Significances are shown as asterisks.

Bahadori et al. [38] examined the personal traits regarding burnout symptoms. Personality traits (neuroticism, extraversion, agreeableness, openness to experience, and conscientiousness) and job stress variables (relationships and roles) showed moderate to high correlations**/***, and there were moderate correlations between neuroticism and job stress demand***. Neuroticism, conscientiousness, and extraversion predicted job burnout.

Guadagni et al. [43] found connections between poor sleep quality and empathy in paramedics, who reported worse quality of sleep and negative images**.

Kirby et al. [42] showed that individuals with adaptive coping strategies had higher spiritual change** and relationships with others* and were associated with personal strength* and lower intrusion. Maladaptive coping strategies were associated with higher scores on avoidance**, hyperarousal, and intrusion.

Nirel et al. [41] investigated the reasons why paramedics worked as paramedics, their work characteristics, and their quality of life. Forty percent of the respondents indicated their reasons as the action and adrenaline from working under pressure, 34% named the ability to help people and save lives, 32% stated the satisfaction and feedback from patients, and 25%pointed out the challenge and working with people. As for the difficult working conditions that were noted, 56% of these incidents involved children, deaths, resuscitations, suicides, and accidents involving children. Sixty percent of the respondents indicated having job satisfaction at all, and 22% indicated having a high level of job satisfaction.

Sterud et al. [40] showed that 18.8% of men and 10.7% of women used alcohol to cope. Depersonalization and emotional exhaustion as symptoms of burnout were associated with higher levels of alcohol problems and consumption. Neuroticism was also related to alcohol problems.

Thielmann et al. [39] examined work-related experience and behavior patterns among the different organizational structures (aid organizations, professional fire departments) in urban and rural regions. Emergency service personnel from aid organizations had more health-promoting patterns, and most employees from professional fire departments (PFDs) had health-risking patterns. The PFDs reported lower distancing ability**, lower experience of success at work***, and lower satisfaction with life** than the aid organizations.

### 3.3. Interpretations of the Studies on Stress and Strain

Twenty-two studies investigated the stress and strain of the emergency service personnel with numerous questionnaires [16,17,18,19,20,21,22,23,24,25,26,27,28,29,30,31,32,33,34,35,36,37]. A total of 26,705 subjects were included. An overview of the included studies is shown in Table 2 and the results are shown in Supplementary File S2. One study examined the combination of behavior/stress and strain and is not repeated here [38]. Three studies were on the combination of well-being/stress and strain [28,35,46], of which one is also described in the well-being section [46].

Alexander et al. [37] determined the mental health factors and occupational factors and examined their relationship to critical incidents. Sixty-nine percent of the subjects stated that they never had enough time to mentally recover between critical incidents. There were, for example, weak negative correlations between personal accomplishment and years of experience, moderate negative correlations between job satisfaction and MBI emotional exhaustion and depersonalization***, moderate negative correlations between organizational satisfaction and depersonalization***, and weak negative correlations between organizational satisfaction and emotional exhaustion**.

Almutairi et al. [36] identified the coping strategies and levels of burnout among EMS. They also experienced high levels of emotional exhaustion and depersonalization and low levels of personal achievement; in addition, the majority of the respondents reported using coping strategies such as talking with colleagues (87.4%), looking forward to being off duty (82.6%), and thinking about the positive benefits of work (81.1%).

Bennett et al. [35] demonstrated that a lower occupational qualification was associated with more stress for incident-related stress*, organizational-related stress, and frequency of potentially traumatic incidents. Mainly, organizational stress had moderate and high correlations with posttraumatic stress disorder (PTSD)**, with an odds ratio of 1.1.

Berger et al. [34] examined the prevalence of PTSD in EMS. The prevalence rate of PTSD was 5.6%; for partial PTSD, the rate was 15%. EMS with PTSD symptoms had more frequent self-reported emotional problems***, more medical visits**, poorer quality of life**, and poorer general health**.

Berth et al. [33] indicated a gratification crisis in 70.7% of EMS. Associations between this gratification crisis and higher ages, lower education levels, higher qualifications, marital status, poorer health status, lower social support, and depression were found.

Braun et al. [32] explored the subjective stress levels and cortisol levels in a comparison between rescue flight days, clinic days, and free days. The cortisol awakening response to stress was higher on rescue flight days than on clinic days or free days, where work experience*** had an impact on this response.

Cash et al. [31] compared the qualifications of the providers of basic life support (BLS) and advanced life support (ALS). Here, the higher qualification (i.e., ALS) offered providers more chronic stress and poorer sleep quality. The same was seen with older subjects rather than younger subjects.

Crowe et al. [30] examined the prevalence of burnout in EMS (8%). The predictors of burnout were time pressure (adjusted odds ratio, AOR: 4.4), waiting to respond in an emergency vehicle rather than a base station (2.3), working 12 or more shifts ≥24 h in the past 30 days (2.3), working 12 or more night shifts in the past 30 days (1.5), and one’s work environment not including a place to exercise (3.0), relax (2.5), or eat (2.8). Job resources such as respect from supervisor or management support (0.2) and control over schedule (0.30) helped to reduce burnout.

Deniz et al. [29] showed that 61.7% of EMS reported that violence occurred during the night shift. Only 10% of the EMS received psychological or legal support. A total of 63.3% of the EMS found administrative directors to be unsupportive in cases of exposure to violence, which led to the experience of fear of violence***. Violence experienced in the workplace is related to emotional exhaustion, depersonalization, and the personal accomplishment of burnout syndrome. No difference in the fear of violence and exposure to violence between females and males was found. The greatest level of fear was reported in the 18–29-year age group.

Hruska and Barduhn [28] and Heringshausen et al. [46] reached the conclusion that EMS had a heightened risk for PTSD and depression relative to other occupational populations. The recovery activities that one engages in may protect these individuals against depression and reveal several dynamic psychosocial factors that aid in understanding features of the work day that contribute to the mental health burden observed among EMS.

Iorga et al. [27] found relationships between work satisfaction, alexithymia, length of service, gender, and the occurrence of burnout syndrome among EMS. Females with high alexithymia scores had higher burnout values*. Those with a length of service over 16 years and low levels of professional satisfaction had higher burnout values***.

Iranmanesh et al. [26] examined the incidence of PTSD among EMS (94%) and factors influencing the PTSD score and compared these data with those of hospital emergency personnel. The authors found a negative correlation of working hours, low interest in work, direct patient contact, and PTSD scores***. Scores were higher for hospital staff than for ambulance service.

Jonsson et al. [25] also identified the prevalence of PTSD (15.2%) and influencing variables in EMS. A total of 61.6% of EMS personnel experienced one or more traumatic events. Higher PTSD scores were found in combination with a lower sense of coherence**, higher age**, working years**, high physical and psychological workload**, and shorter general education.

Khashaba et al. [24] compared the psychosocial stress and related hazards between emergency medical responders and the control group. Compared to the control group, EMS showed more overall job stressors**, a lack of decision control***, poorer communication with their organization**, higher levels of emotional exhaustion***, depersonalization**, and personal achievement***, and a higher PTSD rate***.

Ma et al. [23] conducted a study one month after an earthquake and investigated the risk factors related to posttraumatic stress disorder among the disaster rescue workers. The PTSD prevalence rate was 1.4%; the rate for partial PTSD was 12.7%. Symptoms were re-experience (11.8%), avoidance (12.7%), and hyperarousal (4.7%). EMS with higher levels of partial PTSD were also easily anxious***, perfectionist*, or introvert/socially inactive.

Maguire et al. [22] performed the only international study and focused on acts of violence, using the U.S. as the main focus. In addition to the USA, Australia, the UK, Ireland, New Zealand, Canada, Sweden, Germany, and other countries were investigated. Sixty-five percent of EMS were physically attacked, with males being 1.38 times more likely to be attacked. No significant difference was found regarding nationality. Younger adults experienced more attacks. A total of 40% of the attacks were observed from 04:00 p.m. to midnight; in addition, 90% of the aggressors were patients, and 5% were family members of patients.

Mishra et al. [21] determined the prevalence of PTSD in EMS personnel. Seventy-one percent of EMS patients underwent a traumatic event, and the prevalence of PTSD was 4%. Symptoms were complained of in 81% of EMS such as the following PTSD criteria: exposure (22%), re-experience (27%), avoidance (8%), hyperarousal (26%), and impairment in functioning (29%). Younger people were significantly more likely to be affected than older people. The following coping strategies were used: positive reinterpretation (63%), seeking family and social support (59%), awareness and venting of emotions (46%), and use of alcohol and drugs (10%).

Okada et al. [20] also studied the working conditions and health of EMS. Various physical symptoms were present under physical stress conditions: 66.6% of the respondents reported physical stress (42.6% with symptoms/pain) in the lower back, 36.7% (17.1% with symptoms/pain) in the neck, 27.9% (14.1% with symptoms/pain) in the knees, 24.3% (12.3% with symptoms/pain) in the upper back, and 33.5% (16.4% with symptoms/pain) in the shoulders. Age (older), qualifications as a paramedic, working conditions, and number of dispatches per ambulance were related to the level of mental stress, but paramedics felt more satisfaction with their job than the other EMTs.

Popa et al. [19] investigated the burnout symptoms between different qualifications of EMS. Physicians had the highest scores for emotional exhaustion*** and depersonalization*** compared to paramedics or drivers.

Rybojad et al. [18] focused on the prevalence of PTSD and its risk factors. The prevalence of PTSD of EMS was 40.0% (women 64.3%; men 36.1%), which was more frequent in subjects who had experienced multiple traumatic events.

Soravia et al. [17] also studied PTSD. The prevalence of PTSD was higher in individuals employed in ambulance (15%) and emergency (18%) services than in other rescue workers such as police services (15%) and fire services (8%). The reasons for this outcome could be the higher levels of pressure and stress due to having more emergency calls and close contact with victims. The risk factors for PTSD were dysfunctional coping strategies, high self-efficacy, and work-related trauma, with problem-focused coping strategies being only marginally protective.

Von der Ploeg et al. [16] investigated the acute and chronic job stressors among the EMS and their predictors of health symptoms. A total of 12% of the respondents reported an increase in symptoms of burnout over the last five years. A risk for burnout was observed in 8.6% of the EMS. A lack of social support from colleagues and from supervisors was shown to be associated with most health symptoms.

### 3.4. Interpretations of the Studies on Well-Being

Four studies focused on thematic well-being [44,45,46,47]. An overview of the included studies is shown in Table 3 and the results are shown in Supplementary File S2. One study is described in Table 1 [41]. The studies included 3124 subjects.

Heringshausen et al. [46] examined the levels of life satisfaction, well-being, and compatibility in the private and work lifes of EMS. The results showed that EMS who were in stable relationships and had children reported higher levels of life satisfaction than those who were singles*. Working twenty-four-hour shifts also resulted in higher life satisfaction** and well-being* than working 12-h shifts. Higher levels of life satisfaction*, higher levels of well-being**, and lower levels of private life and work compatibility conflict*** were stated from EMS in regions with smaller populations. Four or more alarm operations resulted in lower levels of life satisfaction*, lower levels of well-being***, and higher levels of private life and work compatibility conflict***.

Sterud et al. [45] measured the prevalence of anxiety and depression symptoms among ambulance personnel and compared this prevalence to the general working population. The level of anxiety symptoms was lower for EMS than for the general population, especially for men*** and women*. The level of depression symptoms was also lower for the EMS than for the general population (for men*). Ambulance personnel contacted general practitioners less often than the general population***.

Sterud et al. [44] focused on suicide risk among EMS and found that 22.8% of EMS reported a lifetime suicidal ideation, with 10.4% of those reporting serious suicidal ideation and 3.1% reporting having previously attempted suicide. No gender difference was found, but there was a higher prevalence of women who ‘wished they were dead’*. Younger EMS seriously considered suicide more often than the older EMS. Strong correlations were shown between the variables of not being married, of a low age, having depression symptoms, having low self-esteem, being bullied at work, and experiencing a lifetime suicidal ideation compared to last year’s ideation***. Only 1.8% of EMFs reported work problems as the only factor of importance for their suicidal ideation.

### 3.5. Evaluation Quality Assessment (EPHPP)

All of the studies were rated as weak, which is problematic in cross-sectional studies. Except for three studies [20,22,29], strong ratings were assigned in the data collection methods. The majority of the studies included confounders, but only a few provided controlled information of the relevant confounders, for example, [16,18,20,22,25,29,30,33,36,39,41,45,46]. In the other studies, the data were not clear. Evidence of the studies is low.

## 4. Discussion

This systematic review examined studies of work-related stress, well-being, and mental health among emergency service personnel and emergency physicians. Thirty-three studies worldwide were included in this review, although most of the studies did not consider emergency physicians. The studies were evaluated according to the EPHPP, which is a quality assessment tool for the quantitative studies dictionary, and were classified as weak according to the guidelines therein.

The studies showed a balancing act between the intrinsic motivation and the consequences of the difficult working conditions of EMS. Paramedics seem to be highly motivated by internal factors, are action-oriented, and highly engaged, among other factors [3]. Nevertheless, an enormous degree of job satisfaction was apparent in spite of the high workloads of the job. In addition to time pressures and the volume of assignments, organizational influences and a lack of support were frequently complained of as strains [16,29,33]. Experiencing violence or traumatic events in ambulance service was remarkably common [25,29]. The results of the studies indicated an increased prevalence of psychological stress factors and possible consequences. However, the studies listed did not take into account the currently existing SARS-CoV-2 pandemic. Recent survey results from EMS in Germany illustrate that the current pandemic has led to an increase in the perceived daily workload [48]. A total of 87.4% of EMS reported an increase in mental stress and 80.6% reported an increase in physical stress during the first, second, and third waves. The perceived workload and level of job dissatisfaction are variables that are currently very highly associated with thoughts of changing jobs [48]. The importance of studies on psychological stress (e.g., stress resulting from everyday guarding and work or the place of operation (city, country)), is currently still limited and will hopefully increase in the future [39]. Leadership behavior and support plays a large role in the perceptions of stress and job satisfaction [16,30,33,49].

Although burnout and PTSD have been examined in several studies, different versions of questionnaires or even completely different questionnaires have been used in these studies. Therefore, no statistical comparisons were made herein. The studies showed increased levels of burnout risk and certain symptoms and a moderate negative correlation between job satisfaction and MBI emotional exhaustion and depersonalization. Regarding PTSD, a wide range of results was found in the different studies (i.e., 1.4% [23] to 40.0% of EMS [18]). The studies showed a high risk for PTSD if one knew or identified with the victim or if one self-experienced trauma (not only work-related trauma). The severity of the victim’s injuries appeared to be less relevant to PTSD outcomes [39]. Long-term stressful working conditions can have a negative impact on the physical and psychological health of the EMS. This is especially important when stress compensation is insufficient [20,50]. Many of the included studies showed high levels of stress, violence experienced, and ultimately PTSD, burnout, or other mental health disorders [16,17,18,19,21,23,25,26,27,28,29,30,33,34,35,36,37]. Job satisfaction and working conditions also have a great importance in the development of emotional exhaustion and depersonalization [51]. Age, and therefore, work experience are not necessarily protective against stress. The results taking gender into account are not consistent [20,25]. Males showed more emotional exhaustion and depersonalization in MBI than females, but females with high alexithymia scores had higher burnout values [27]. Unfortunately, the studies are not directly comparable with each other since a large number of different questionnaires were used. It is also unclear whether all job titles are the same in all countries. Care was taken to ensure that the typical job titles were listed and the ambulance service was described. Evidence of the studies was low. This may be because cross-sectional studies usually have low evidence. Nevertheless, these studies can still be trendsetting in their nature. Growing old healthily and with the motivation to continue working in emergency ambulance services is desirable, but only very few rescue workers are currently able to achieve these goals. Age and work experience do not necessarily appear to be protective against psychological stress and its consequences [20,34]. This may be because the risk of experiencing traumatic events and acts of violence increases with years of work in rescue services. Future concepts of health promotion and prevention that focus on the EMS as an individual in their entirety are necessary and at the center of the focus of attention. The same is applicable to emergency physicians, who, overall, have received limited attention in the literature. It is possible that not all studies were included because studies that were not open access, could not be obtained through interlibrary loans, or for which there had been no response from the authors upon request could not be included.

Since the frequency of alarm operations and technical protective measures can only be realized to a limited extent (e.g., locking of vehicles, bringing two-way radios to operation locations), the implementation of organizational (e.g., providing instructions, conducting supervision, providing crisis intervention for staff) and personal protective measures (e.g., allowing supervision, trainings) plays a major role in the EMS profession.

A higher level of professional qualification appears to have a positive effect on health despite one also having higher levels of responsibility [16,34]. Often, even small changes have a satisfactory effect. Furthermore, the employees’ wishes should be made known, and work schedules should be drawn up at an early stage and take the employees’ personal wishes into account [21]. Respect from supervisors and support from management are also factors related to satisfaction [21]. Strengthening the intrinsic motivation of EMS should be a major focus of health promotion and prevention interventions. A meta-analysis showed that intrinsic motivation is a moderate to strong predictor of performance [52].

## 5. Conclusions

Given that they safeguard the patient care of the population, emergency medical services personnel are exposed to special workloads. Therefore, EMS should be particularly protected, while also considering the current shortage of skilled workers, which varies both nationally and internationally. The implementation of health promotion measures in the workplace should be carried out while also raising awareness of these measures among the EMS. It also seems useful to conduct future studies that examine, for example, interventions of preventive measures and their outcome. Strengthening the motivation of employees or the training of leaders is recommended. Attention should also be given to implementing all of the technical measures to reduce stress (e.g., locking of ambulance vehicles in the case of violent patients or relatives, or the use of electro-hydraulic stretchers).

## Figures and Tables

**Table 1:** An overview of the included studies with thematic of behavior (the results are shown in Supplementary File S2).

Author ^t^ Country	Subject Number; Gender; Age (Mean ± SD, Years)	Questionnaire Task
**Bahadori ^#^, 2019 [38]** Iran	308 EMS; 100% ♂; 30.0 ± 5.4	HSE, MBI NEO-FFI
**Guadagni, 2018 [43]** Canada	41 (12 PM, 13 PM trainees, 16 CG); 51% ♀, 49% ♂; PM 33.2 ± 5.47; PM trainees 25.8 ± 6; CG 23 ± 7.1	PSQI, STAI, BDI, Davidson Trauma Scale, Emotional Empathy task
**Kirby, 2011 [42]** Australia	118; 34% ♀, 66% ♂; 37.0 ± 10.5	PTGI, IES-R, R- COPE
**Nirel ^√^, 2008 [41]** Israel	328 PM; 13% ♀, 87% ♂; 22 to ->40	n.s. work overload, satisfaction with choice of profession, job satisfaction, burnout measure, satisfaction with the choice of profession
**Sterud, 2007 [40]** Norway	1096; 23% ♀, 77% ♂, 36.8 ± 9.3 (18–66)	AUDIT, Drinking to cope, MBI, Job Stress Survey, BCI
**Thielmann, 2021 [39]** Germany	276 (125 AOT, 70 PFD, 81 AOC); 5.4% ♀, 94.6% ♂; 39.3 ± 8.0	AVEM

Notes. ^t^ First authors named alphabetically. ^#^ combination stress/strain and behavior. ^√^ combination behavior/well-being ♀ = female ♂ = male. PM = paramedic, CG = control group, PTSD = Posttraumatic stress disorder PSQI = Pittsburgh Sleep Quality Index, STAI = State-trait anxiety inventory, BDI = Beck depression inventory, PTGI = Posttraumatic Growth Inventory, IES-R = Impact of Events Scale-Revised, R-Cope = R-COPE Inventory, AVEM = Work-related behavior and experience patterns, NEO-FFI = revised Costa and McCrae NEO Five-Factor Inventory, BCI = Basic Character Inventory, SD = standard deviation.

**Table 2:** An overview of the included studies with the thematic of stress and strain (the results are shown in Supplementary File S2).

Author ^t^ Country	Subjects Number; Gender; Age (Mean ± SD, Years)	Questionnaire Task
**Alexander, 2001 [37]** UK	110 (40 PM, 70 EMT); 14% ♀, 86% ♂; 30–39	GHQ-28, IES, MBI, HS, PMI, CMC
**ALmutairi, 2020 [36]** Saudi Arabia	270 EMS; 100% ♂; 30–38	MBI, CMC
**Bennett ^≈^, 2005 [35]** UK	617 (194 EMT, 380 PM, 43 n.n.); 15% ♀, 83% ♂; 2% n.r. 39,6 ± 10.6	AWSQ, PDS, HADS, CAQ
**Berger, 2007 [34]** Brazil	234 (29 % EP); 23% ♀, 77% ♂; 32.6 ± 8.1	PCL-L, SF-36
**Berth, 2018 [33]** Germany	82 EMS; 27% ♀, 73% ♂; 32.8 ± 10.9	ERI, SOP-2, OSSS, MOB-K, PHQ-4
**Braun, 2021 [32]** Germany, Switzerland	40 EP; 30% ♀, 70% ♂; 40.7 ± 6.5	CAR, HRV, TICS, PSS
**Cash, 2020 [31]** USA	17,522 EMS; 27% ♀, 73% ♂; 34.2 ± 0.1	PSQI, PSS, Chronic burden scale
**Crowe, 2019 [30]** USA	1721 EMS; 26% ♀, 74% ♂; 19–75	CBI, Questionnaire of job demands and resources proposed by Demerouti et al. as a foundation,
**Deniz, 2015 [29]** Turkey	120 ambulance staff; 47% ♀, 53% ♂; 29.5 ± 6.5	MBI, exposure to violence
**Hruska ^≈^, 2021 [28]** USA	79 EMS; 49% ♀, 51% ♂; 30.7 ± 9.4	SF-PCL-5, MHI-d, Checklist for occupational stressors, Consensus Sleep diary (CSD), social conflict, 3-items of the Brief COPE, PEAT, ISSB, Perceived Prosocial Impact
**Iorga, 2015 [27]** Romania	122 EP, EMS; 51% ♀, 49% ♂; age n.r.	TAS-20, MBI, Job Satisfaction Questionnaire
**Iranmanesh, 2013 [26]** Iran	400 (150 PM, 250 HEP); 43% ♀, 57% ♂; PM: 29.3 ± 6.0 EMS: 31.2 ± 6.3	M-PTSD
**Jonsson, 2003 [25]** Sweden	362 EMS; 21% ♀, 79% ♂; 38.4 ± 7.9	SOC, IES-15, PTSS-10, traumatic events
**Khashaba, 2014 [24]** Egypt	280 (140 EMT, 140 CG); 100% ♂; 37 ± 9.4	MBI, BDI, DTS
**Ma, 2020 [23]** Taiwan	447 EMT; 6.5% ♀, 93.5% ♂; 38.8 ± 8.4	PCL
**Maguire, 2018 [22]** US international	1.747; 31% ♀, 69% ♂; ≤25 to >55	n.s.
**Mishra, 2010 [21]** USA	101 EMS; 43% ♀, 57% ♂; 35–54	PCL-C
**Okada, 2005 [20]** Japan	1551 PM/EMT; 1% ♀, 99% ♂; 34.6 ± 8.2	n.s. physical stress, mental stress (environment, worksite, symptoms)
**Popa, 2010 [19]** Romania	258 PM, 1.395 ambulance drivers, 243 EP; n.r.; n.r.	MBI-HSS
**Rybojad, 2016 [18]** Poland	100 EMS; 14% ♀, 86% ♂; 33.6 ± 9.3	IES-R
**Soravia, 2021 [17]** Switzerland	97; 43% ♀, 57% ♂; 38.7 ± 10.0	PTSS, GHQ-12, BSI, PSES, Coping strategies, Suicidal ideation
**van der Ploeg, 2003 [16]** Netherland	123; 14% ♀, 86% ♂; 39.8 ± 7.1	QEAW, IES, CIS, MBI

Notes. ^t^ First authors named alphabetically. ♀ = female ♂ = male. ^≈^ Combination with thematic wellbeing. PM = paramedic, EMS = Emergency medical services professionals (included different types of professional qualification), EMT = Emergency Medical Technicians, n.n. = nomen nominandum/unknown, EP = emergency physician, HEP = Hospital emergency personnel, CG = Control group, n.s. = not standardized, n.r. = not reported, SD = standard deviation, AOR= Adjusted OR. GHQ = General Health Questionnaire, IES/IES-R/IES-15 = The Impact of Event Scale/-Revised, MBI/MBI-HSS = Maslach Burnout Inventory/MBI-Human Services Survey, HS = Hardiness Scale, PMI = Pressure Management Indicator, CMC = Coping Methods Checklist, HSE = Health and Safety Executive Job Stress Questionnaire, AWSQ = Ambulance Work Stressors Questionnaire, PDF = Posttraumatic diagnostic scale, HADS = Hospital anxiety and depression scale, PCL-C = Posttraumatic stress disorder checklist-civilian version, SF-36 = short form Health survey-36, ERI = Effort-Reward-Imbalance-Questionnaire, SOP-2 = Optimism-Pessimism-2 Scale (German Skala Optimismus-Pessimismus-2), OSSS = Oslo Social Support Scale, MOB-4 = Scale bullying intensity of colleagues (German Skala Mobbingintensität der Kolleginnen und Kollegen), PHQ-4 = Patient Health Questionnaire 4, CAR = Cortisol awakening response, HRV = Heart Rate Variability, TICS = Trier inventory for chronic stress, PSS = Perceived stress scale, PSQI = Pittsburgh Sleep Quality Index, CBI = Copenhagen Burnout Inventory, SF-PCL-5 = 4-item Short-Form PTSD Checklist-5, MHI-d = 3-item Mental Health Inventory-depression scale, CSD = Consensus Sleep diary, PEAT = Pittsburgh Enjoyable Activities Test, ISSB = Inventory of socially supportive behaviors, TAS-20 = Toronto Alexithymia Scale, M-PTSD = Mississippi scale for posttraumatic stress disorder, SOC = Sense of Coherence Scale, PTSS-10 = Post Traumatic Symptom Scale, BDI = Beck depression inventory, DTS = Davidson Trauma scale for PTSD, PCL = Post traumatic stress disorder checklist, BSI = Brief Symptom Inventory, PSES = General Perceived Self-Efficacy Scale, AUDIT = Alcohol Use Disorders Identification Text, QEAW = Questionnaire on the Experience and Assessment of Work, CIS = Checklist of Individual Strength.

**Table 3:** An overview of the included studies with the thematic of well-being (the results are shown in Supplementary File S2).

Author ^t^ Country	Subjects Number; Gender; Age (Mean ± SD, Years)	Questionnaire Task
**Gayton, 2012 [47]** Austria	219; 11% ♀, 89% ♂; age groups (mean 22–43)	CD-RISC, SFWL, GHQ-28
**Heringshausen ^∞^, 2010 [46]** Austria/Germany	545 EMS; 11% ♀, 89% ♂; 38.0 ± 9.6	WHO-5, SWLS, WFC
**Sterud, 2008a [45]** Norway	1180 EMS, 31,987 CG; 23% ♀, 77% ♂; 36.8 ± 9.3	HADS, Karolinska Sleep Questionnaire, SHC, Need for Recovery after Work Scale
**Sterud, 2008b [44]** Norway	1180 EMS; 23% ♀, 77% ♂; 36.8 ± 9.3	HADS, BCI, RSES, JSS, MBI-HSS, Paykel’s Suicidal Feelings in the General Population questionnaire, SHC

Notes. ^t^ First authors named alphabetically. ♀ = female ♂ = male. ^∞^ Combination with stress and strain. PM = paramedic, at = ambulance technicians, EMS = Emergency medical services professionals (included different types of professional qualification), EMT = Emergency Medical Technicians, n.n. = nomen nominandum/unknown, EP = emergency physician, HEP = Hospital emergency personnel. WHO-5 = Well-Being Index, SWLS = Satisfaction With Life Scale, WFC = Privacy Conflict, CD-RISC = Connor–Davidson Resilience Scale, SFWL = Satisfaction With Life Scale, GHQ-28 = General Health Questionnaire, HADS = The Hospital Anxiety and Depression scale, SHC = Subjective Health Complaint, BCI = Basic Character Inventory, RSES = Rosenberg Self-Esteem Scale, JSS = Job Satisfaction Scale, MBI = Maslach Burnout Inventory—Human Services Survey, WFC = Scala Work-(Family) Privacy, Gayton and Lovell [47] determined the association between resilience and years of work experience, general health, and well-being. Key points of the study included that resilience increases work experience* and is dependent on age**. The study found a moderate correlation between resilience and general health or well-being**.

## Data Availability

The data can be requested from the authors.

## Supplementary Materials

The following supporting information can be downloaded at: https://www.mdpi.com/article/10.3390/ijerph19116660/s1, Supplementary File S1: The abbreviations and explanations of the questionnaires used in the included studies. Supplementary File S2: The results of the included studies.
