# Supplementary material for: Analysis of Work Related Factors, Behavior, Well-Being Outcome, and Job Satisfaction of Workers of Emergency Medical Service: A Systematic Review
Source: Int J Environ Res Public Health. 2022 May 30;19(11):6660. doi: 10.3390/ijerph19116660 (PMC9180048; doi:10.3390/ijerph19116660)
Supplement: Supplementary file 1 [file ijerph-19-06660-s001.zip › Supplementary File S1.pdf]

Abbreviation	Name	Measure	Items
Table 1: Questionnaire behavior			
R-COPE	R-COPE Inventory		
NEO-FFI	revised Costa and McCrae NEO Five-Factor Inventory	Personality traits	Self-description of neuroticism, extraversion, openness, agreeableness, consciousness
STAI	state-trait anxiety inventory	State anxiety (about an event) and trait anxiety (personality trait) separately	Strength of anxiety feelings; State-anxiety as a current state of anxiety (fear, nervousness, discomfort, arousal of autonomic nervous system); Trait anxiety as stable aspects of anxiety (stress, worry, discomfort daily)
PSQI	Pittsburgh Sleep Quality Index	Quality of sleep and disturbance	Self-description of subjective sleep quality, sleep latency, sleep duration, habitual sleep efficiency, sleep disturbances, use of sleeping medication, and daytime dysfunction over 1 month
BDI	Beck Depression Inventory	Severity of depression	Self-description of affective, cognitive, somatic, and vegetative symptoms and attitudes associated with depressive disorders
AVEM	Arbeitsbezogenes Verhaltens-und Erlebensmuster	Work-related Behavior and Experience Pattern	Effect of internal factors like individual behavior and experience patterns on work behavior
PTGI	Posttraumatic Growth Inventory	Positive outcomes after extremely stressful and potentially traumatic events	New possibilities, Relating to others, Personal strength, Spiritual change, Appreciation of life
	Job Stress Survey	Organizational stressors	Job pressure, lack of support
Table 2: Questionnaire stress and strain			
HSE	Health and Safety Executive	Working conditions	Performance of an organization in demands, control, manager support, peer support, relationships, role and change at work
MBI	Maslach Burnout Inventory	Burnout score	Emotional exhaustion, depersonalization, personal accomplishment
HADS	Hospital anxiety and depression scale	Depression symptoms and anxienty	Non-physical symptoms of anxiety and depression
PCL-C	posttraumatic stress disorder checklist-civilian version	PTSD symptoms	Symptomatic responses of PTSD related symptoms
TICS	trier inventory for chronic stress	Chronic stress	Work Overload, Social Overload, Pressure to Perform, Work Discontent, Excessive Demands from Work, Lack of Social Recognition, Social Tensions, Social Isolation, and Chronic Worrying

PSS	Perceived stress scale	Individual stress levels	Feelings and thoughts during the last month
SF-36	short form Health survey-36	General health status	vitality, physical functioning, pain, general health, physical role, social function, emotional role, and mental health
AUDIT	Alcohol use disorder identification test	Alcohol use	Screening for unhealthy alcohol use disorder
PSQI	Pittsburgh Sleep Quality Index	Quality of sleep and disturbance	Self-description of subjective sleep quality, sleep latency, sleep duration, habitual sleep efficiency, sleep disturbances, use of sleeping medication, and daytime dysfunction over 1 month
SF-PCL-5	Short-Form PTSD Checklist-5	PTSD symptoms	Self-description of PTSD symptoms used for diagnosis, screening and monitoring during and after treatment
MHI-5 (MHI-d= short version)	Mental Health Inventory-depression scale	Mental health factors	anxiety, depression, behavioral control, positive affect, and general distress
CSD	Consensus Sleep diary	Sleep characteristics	Sleep disorders especially Insomnia
PEAT	Pittsburgh Enjoyable Activities Test	Leisure activities	Frequency of involvement in enjoyable activities
ISSB	Inventory of socially supportive behaviors	Social support	Frequency the social environment has helped with certain tasks during the past 4 weeks
TAS-20	Toronto Alexithymia Scale	Alexithymia	Ability to understand, process, and communicate emotions and feelings
M-PTSD	Mississippi scale for post-traumatic stress disorder	Combat-related PTSD	Frequency of PTSD symptoms and changes in life after serving in the military
BDI	Beck-depression Inventory	Severity of depression	Self-description of affective, cognitive, somatic, and vegetative symptoms and attitudes associated with depressive disorders
DTS	Davidson Trauma scale for PTSD	PTSD symptoms	Self-description of frequency and severity of PTSD symptoms, helps to make the diagnosis of PTSD
PCL	Post-traumatic stress disorder checklist	PTSD symptoms	Changes in existing symptoms and the addition of new symptoms
GHQ-28	General Health Questionnaire	Psychological well-being and psychiatric disorders	Depression, Psychological distress, Anxiety, Social impairment, Hypochondriasis
IES-15	The Impact of Event Scale	Amount of subjective distress in response to trauma; discriminate between trauma related and non-trauma related symptoms	Experiences of avoidance (numbing of responsiveness, avoidance of feelings, situations, ideas) and intrusion (intrusive thoughts, nightmares, intrusive feelings and imagery)
HS	Hardiness Scale	Hardiness/resistance	control, communication, and challenge

PMI	Pressure Management Indicator	Factors involved in stress process and pressure profile	Outcome scale, sources of pressure scale, behavior and coping variables
CMC	Coping Methods Checklist	Coping strategies	Helpfulness of various strategies to deal with chronic stress
ERI	Effort-Reward-Imbalance-Questionnaire	Effort-reward imbalance	effort (physical load), reward (Esteem, Promotion, security), and over-commitment
SOP-2	Optimismus-Pessimismus-2 Skala	Psychological characteristic optimism-pessimism	Optimistic thoughts, pessimistic thoughts
OSSS	Oslo-3-Social-Support-Scale	Social functioning, predictor of mental health	number of people the respondent feels close to, interest and concern shown by others, ease of obtaining practical help from others
MOB-K	Skala Mobbingintensität der Kolleginnen und Kollegen	bullying intensity of colleagues	Subjective intensity of feeling bullied at work
PHQ-4	Patient Health Questionnaire 4	Psychological distress	Anxiety and depression
CBI	Copenhagen Burnout Inventory	Burnout	personal burnout, work-related burnout, client-related burnout
SOC	Sense of Coherence Scale	comprehensibility, manageability, and meaningfulness	View on life and use of resistance resources to maintain and develop health
PTSS-10	Post Traumatic Symptom Scale	Post-traumatic Symptoms	Applying of statements related to reactions to a particular event
IES-R	Impact of Events Scale-Revised	PTSD symptoms	Subjective distress and annoyance during the past 7 days, used for a preliminary diagnosis
MBI-HSS	Maslach Burnout Inventory-Human services survey	Burnout in certain professions	Emotional Exhaustion, Depersonalization, Personal Accomplishment
AWSQ	Ambulance Work Stressor Questionnaire	Degree of stress associated with stressors within ambulance work	incident-related stressors (dealing with incidents that involve children, burns patients, handling dead bodies), organization-related stressors (tension with colleagues, going to incidents that were false alarms, conflicts between work and home)
PDF	Posttraumatic diagnostic scale	severity score and a diagnostic categorization for PTSD	re-experiencing traumatic memories, attempts at avoidance, and arousal
CAQ	Cognitive Appraisal Questionnaire	Cognitive and emotional response to an event that potentially triggers their symptoms	presence of dissociative symptoms at the time of a work-related traumatic event

CAR	Cortisol awakening response	Psychological stress	Change in cortisol concentration in the first hour after waking up from sleep
HRV	Heart Rate Variability	Physical impact of emotional arousal	Variation in the time interval between heart beats
BSI	Brief Symptom Inventory	Psychological distress and psychiatric disorders	Global Severity Index (GSI), Positive Distress Index (PSDI), Positive Symptom Total (PST)
PSES	Perceived Self-Efficacy Scale	Self-efficacy	general optimistic competence expectation
QEAW	Questionnaire on the Experience and Assessment of Work	Chronic work-related stressors	Poor communication, Insufficient financial reward, High emotional demands, Lack of information, Lack of support from colleagues, Lack of support from supervisor, Physical strains, Lack of job autonomy
CIS	Checklist of Individual Strength	Fatigue	subjective fatigue, concentration, motivation, physical activity
	Chronic burden scale	Presence and severity of ongoing stress	one's own health problems, health problems of close others, job, or ability to work, relationships, finances
	Checklist for occupational stressors	Occupational stressors	chronic, routine work challenges, high impact incidents
	Perceived Prosocial Impact	Perceived Prosocial impact	Awareness of job impact on others
Table 3:			
MBI	Maslach Burnout Inventory	Burnout score	Emotional exhaustion, depersonalization, personal accomplishment
HADS	Hospital anxiety and depression scale	Depression symptoms and anxiety	Non-physical symptoms of anxiety and depression
SHC	Subjective Health Complaint	Subjective experience of health	Nature of complaints and frequency of somatic complaints
JSS	Job Satisfaction Scale	General organizational stressors	Perceived severity and frequency of stress in situations that are encountered in a wide variety of occupations
GHQ-28	General Health Questionnaire	Psychological well-being and psychiatric disorders	Depression, Psychological distress, Anxiety, Social impairment, Hypochondriasis
CD-RISC	Connor–Davidson Resilience Scale	Resilient characteristics	perceived adaptive strategies in stressful situations
SFWL	The Satisfaction with Life Scale	General health and well-being	a person's global judgment of life satisfaction and change in subjective well-being
WHO-5	Well-Being Index	Well-being, Depression-screening	Subjective quality of life: Positive mood, vitality, general interest
SWLS	Satisfaction With Life Scale	Life satisfaction (Part of COPSOQ)	Satisfaction with life as a whole

WFC	Skala Work-(Family) Privacy Conflict	Work and family inter-role conflict (Part of COPSOQ)	Interference and problems between family and work
BCI	Basic character Inventory	Personality	Neuroticism, extroversion, control
RSES	Rosenberg Self-Esteem Scale	Self-esteem	Positive and negative attitude of a person towards themselves
	Karolinska Sleep Questionnaire	Sleep disturbance	difficulties falling asleep, repeated awakenings, premature awakening, disturbed/restless sleep
MBI-HSS	Maslach Burnout Inventory-Human services survey	Burnout in certain professions	Emotional Exhaustion, Depersonalization, Personal Accomplishment
	Need for Recovery after Work Scale	Need for Recovery after work	Need for relaxation after work, intensity of worn-out feeling after work
	Paykel's Suicidal Feelings in the General Population questionnaire	prevalence of suicidal ideation and suicide attempts	Influences, nature, and presence of suicidal thoughts
Table 4: other			
SSS	Stanford sleepiness Scale	Subjective assessment of sleepiness	Self-rated level of sleepiness at the time of the evaluation
TEQ	Toronto empathy Questionnaire	Affective empathy	Level of self-reported empathy
RMET	Reading the Mind in Eyes test	Cognitive empathy, Theory of mind	Ability to identify and understand someone else's mental state
PSS-SR	PTSD Symptom Scale Interview	Symptoms of PTSD	Severity, frequency, re-experiencing, avoidance, arousal of PTSD symptoms
PDS	Posttraumatic diagnostic scale	PTSD Symptom severity during the last month	Frequency and severity of symptoms
PAR	Physician achievement review (adapted version)	performance	Clinical competency, psychosocial management of patients, patient interaction, professional self-management, consultation communication, collegiality, coworker communication, Phone communication, Information for Patients, personal communication, office staff, physical office, appointments
PVT	Psychomotor vigilance task	Identify Sleepiness by measuring alertness, psychomotor skills, false responding	Reaction time, number of times a stimulus is not noted
MINI	Mini-International Neuropsychiatric Interview	Major psychiatric disorders	Assessment of the 17 most common disorders in mental health
CAPS	Clinician-Administered PTSD Scale for DSM-5	PTSD	Make a diagnosis, determine a lifetime diagnosis, and evaluate

			PTSD symptoms over the previous week
WHOQOL-BREF	world health organization quality of life- BREF	Quality of life	Position in life, culture and value system of their environment, goals, expectations, standards, and concerns
